# An extension to a statistical approach for family based association studies provides insights into genetic risk factors for multiple sclerosis in the *HLA-DRB1 *gene

**DOI:** 10.1186/1471-2350-10-10

**Published:** 2009-02-04

**Authors:** Sreeram V Ramagopalan, Roisin McMahon, David A Dyment, A Dessa Sadovnick, George C Ebers, Knut M Wittkowski

**Affiliations:** 1 Department of Clinical Neurology, University of Oxford, The West Wing, The John Radcliffe Hospital, Oxford, OX3 9DU, UK; 2 Wellcome Trust Centre for Human Genetics, University of Oxford, Roosevelt Drive, Oxford, OX3 7BN, UK; 3 Department of Medical Genetics and Faculty of Medicine, Division of Neurology, University of British Columbia, G920, Detwiller Pavilion, VCHA – UBC Hospital, 2211 Wesbrook Mall, Vancouver, British Columbia, V6T 2B5, Canada; 4 Center for Clinical and Translational Science, The Rockefeller University, 1230 York Ave Box 322, New York, NY 10021, USA

## Abstract

**Background:**

Multiple sclerosis (MS) is a complex trait in which genes in the MHC class II region exert the single strongest effect on genetic susceptibility. The principal MHC class II haplotype that increases MS risk in individuals of Northern European descent are those that bear HLA-DRB1*15. However, several other *HLA-DRB1 *alleles have been positively and negatively associated with MS and each of the main allelotypes is composed of many sub-allelotypes with slightly different sequence composition. Given the role of this locus in antigen presentation it has been suggested that variations in the peptide binding site of the allele may underlie allelic variation in disease risk.

**Methods:**

In an investigation of 7,333 individuals from 1,352 MS families, we assessed the nucleotide sequence of *HLA-DRB1 *for any effects on disease susceptibility extending a recently published method of statistical analysis for family-based association studies to the particular challenges of hyper-variable genetic regions.

**Results:**

We found that amino acid 60 of the *HLA-DRB1 *peptide sequence, which had previously been postulated based on structural features, is unlikely to play a major role. Instead, empirical evidence based on sequence information suggests that MS susceptibility arises primarily from amino acid 13.

**Conclusion:**

Identifying a single amino acid as a major risk factor provides major practical implications for risk and for the exploration of mechanisms, although the mechanism of amino acid 13 in the *HLA-DRB1 *sequence's involvement in MS as well as the identity of additional variants on MHC haplotypes that influence risk need to be uncovered.

## Background

Multiple sclerosis (MS) is a common inflammatory disease of the central nervous system characterized by myelin loss, axonal pathology and progressive neurological dysfunction [1]. The aetiology of MS is unknown, but it is clear that both genetic and environmental components are important [2].

The only consistent genetic association with MS in Northern Europeans had been with extended MHC haplotypes especially those containing HLA-DRB1*1501 [3]. Recently, the interleukin 7 receptor (*IL7R*) interleukin 2 receptor (*IL2R*), ecotropic viral integration site 5 (EVI5) [4] and kinesin family member 1B (*KIF1B*) [5] genes have been shown to be additional MS susceptibility loci [3,6,7]. However, the MHC is the largest contributor to MS risk [3].

The association between MS and MHC class II has been fine mapped to the extended haplotype HLA-DQA1*0102-DQB1*0602-DRB1*1501-DRB5*0101 [8]. Intense linkage disequilibrium within the MHC has prevented the exact susceptibility locus from being identified. Analysis of the MHC region with a large number of markers as well as classical typing show evidence for the involvement of the class II region only [9,10]. The paradigm is more complex than one in which the HLA-DRB1*15 allele acts solely to increase MS risk. Our previous investigations have shown that HLA-DRB1*15 and HLA-DRB1*17 bearing haplotypes increase risk of MS, while HLA-DRB1*14 and HLA-DRB1*11 bearing haplotypes are protective [11,12]. Additionally, HLA-DRB1*10, DRB1*01, and DRB1*08 interact with HLA-DRB1*15 to influence disease risk [11,12].

MHC class II molecules present antigen to CD4^+ ^T helper cells and are integral to successful maintenance of self tolerance by the immune system and the adaptive immune response to invading pathogens [13]. Each *HLA-DRB1 *allele forms, by the presence of defined amino acid anchors, a number of specific pockets comprising a peptide binding groove [14]. Different *HLA-DRB1 *alleles may thus have different binding affinities for disease-related peptides as determined by their protein sequence, subsequently influencing composition of T cell repertoires, ultimately resulting in *HLA-DRB1 *alleles having varying effects on disease risk. However, protein sequence analysis has failed to provide clarity. Class II alleles in MS patients are structurally no different to those in healthy controls [15]. While some studies have suggested that variable residues in the DR beta chain may determine MS susceptibility [16-18], others found no evidence that MS pathogenesis is mediated by allele overlapping antigen binding sites [19]. However, these studies were based on a relatively small number of individuals and, thus, may have been underpowered to detect any relevant effects [11,12]. More recently, Barcellos *et al. *[20], by aligning the protein sequences of HLA-DRB1*1501, *1503, *1701, *0401, *0801, and *0803 with that of HLA-DRB1*140101, *140102, *140103, and *1404 have suggested that the amino acid at position 60 of the *HLA-DRB1 *protein sequence determines the effect of a *HLA-DRB1 *allele on MS susceptibility. This model however does not take into account other *HLA-DRB1 *resistance alleles, notably HLA-DRB1*11, *10 and *01 [12].

The *HLA-DRB1 *gene is unusual in that many loci in the coding sequence can have any one of the four nucleotides depending on the allelotype. Thus, empirical studies were difficult, first, because such variability requires large sample sizes, but – even more importantly – because most traditional statistical methods are limited to the more typical case of bi-allelic loci. We present here the largest systematic investigation to date and an extended more sensitive statistical approach, which, for the first time, will allow us to determine empirically whether or not the nucleotide or protein sequence of *HLA-DRB1 *can account for allelic susceptibility to MS.

## Methods

### Particpants

All participants in the study were ascertained through the ongoing Canadian Collaborative Project on the Genetic Susceptibility to MS (CCPGSMS), for which the methodology has been previously described [21].

### Genotyping

Total genomic DNA, extracted from whole blood as part of the CCPGSMS, was used to type *HLA-DRB1 *alleles by an allele-specific PCR amplification method [22]. All genotypes were generated blind to pedigree structure and disease status of the individual. Initially, *HLA-DRB1 *alleles were classified into 10 categories, HLA-DRB1*01 to HLA-DRB1*10. Four of them were then subdivided, *05 into *11/12, *06 into *13/14, *02 into *15/16, and *03 into *17/18. Since then, these "two-digit" genotypes have been refined by adding two or four more digits. The first two digits describe the type, which corresponds to the serological antigen carried by an allelotype. The third and fourth digits are used to list the allele subtypes, numbers being assigned in the order in which the DNA sequences were determined. Alleles whose numbers differ in the first four digits must differ in one or more nucleotide substitutions that change the amino acid sequence of the encoded protein. Alleles that differ only by synonymous nucleotide substitutions within the coding sequence are distinguished by the use of the fifth and sixth digits.

In our population, 24 PCRs were carried out to amplify allelotypes corresponding to alleles HLA-DRB1*01, HLA-DRB1*04, HLA-DRB1*07, HLA-DRB1*08, HLA-DRB1*09, HLA-DRB1*10, HLA-DRB1*11, HLA-DRB1*12, HLA-DRB1*13, HLA-DRB1*14, HLA-DRB1*15, HLA-DRB1*16, HLA-DRB1*17, and HLA-DRB1*18. Each *HLA-DRB1 *genotype was scored twice by independent observers.

#### Sequence Information

*HLA-DRB1 *allele sequence information was obtained from the Immunogenetics database of the European Bioinformatics Institute [23], whose nucleotide and amino acid numbering scheme will be used here.

### Statistical Analysis

For terrestrial life forms, the number of alleles per single nucleotide polymorphism (SNP) is limited to five: A, C, G, T, and X (deletion), although only two alleles have been seen for most human SNPs. For *HLA-DRB1 *and *HLA-DRB2 *SNPs, however, up to 4 and 5 nucleotides, respectively, have been observed. As proven in [24], the informative data for each bi-allelic parental mating type (PMT) can be organized into three strata, with two informative filial constellations each:

**Mating**            **Offspring Genotypes**

   A.A~A.C         A.A   A.C

   A.C~A.C         A.A   C.C (A.C children are non-informative)

   A.C~C.C         A.C   C.C

   ...

   T.T~T.X         T.T   T.X

   T.X~T.X         T.T   X.X (T.X children are non-informative)

   T.X~X.X         T.X   X.X

With three alleles, only two constellations need to be considered:

**Mating**            **Offspring Genotypes**

   A.A~C.G         A.C      A.G

   A.C~A.G         A.A   A.C   A.G   C.G

   ...

   X.X~T.G         G.X      T.X

   X.G~X.T         G.T   G.X   T.X   X.X

With four or five alleles, each stratum contains four informative filial genotypes, e.g.:

**Mating**            **Offspring Genotypes**

   A.C~G.T         A.G,   A.T   C.G   C.T

To determine, whether a particular allele confers risk, children with this allele need to be 'paired' within the same PMT stratum with children having the same other allele. For bi-allelic PMTs, this leaves three informative mating types per combination of alleles, each with two informative filial genotypes [24]. For tri-allelic PMTs, two situations may arise. One homozygous parent, also yields only two filial genotypes, while two heterozygous parents, yield strata with four filial genotypes. For quad-allelic PMTs, both parents are necessarily heterozygous yielding, again, four possible filial genotypes.

After excluding non-informative children, all filial genotypes within a PMT stratum have the same expectation under the null hypothesis. Thus, for the special case of bi-allelic parents, it has been suggested [25] that the sign/McNemar test for exact ties be applied [26], i.e. to count how often either of the genes were transmitted, irrespective of the PMT. Stratification [24] yields a test statistic based on counts representing independently observed events (cases born, rather than alleles transmitted, which are subject to identical genetical and environmental confounders). Here, the need for stratification is even more apparent than for bi-allelic parents [27-29], as there are, for instance, twice as many A alleles to be expected among the children of A.C~A.G parents than either C or G alleles.

When developing a test suitable for multi-allelic loci, it is useful to note that the sign test can be written in the two equivalent forms given at the ends of the following equation:

$$\frac{{\left(a-b\right)}^{2}}{a+b}=\frac{4{\left(\frac{a}{2}-\frac{b}{2}\right)}^{2}}{a+b}=\frac{2{\left(a-\frac{a+b}{2}\right)}^{2}}{\frac{a+b}{2}}=\frac{{\left(a-\frac{a+b}{2}\right)}^{2}}{\frac{a+b}{2}}+\frac{{\left(b-\frac{a+b}{2}\right)}^{2}}{\frac{a+b}{2}}=\frac{{\left(a-\frac{a+b}{2}\right)}^{2}}{\frac{1}{2}\frac{a+b}{2}}$$

As $\left(a-b\right)/\sqrt{a+b}\sim_{as.}\chi_{1}^{2}$ and the "standardized residuals" $\left(a-\frac{a+b}{2}\right)/\sqrt{\frac{1}{2}\frac{a+b}{2}}$ and $\left(b-\frac{a+b}{2}\right)/\sqrt{\frac{1}{2}\frac{a+b}{2}}$ are symmetric, each follows asymptotically a standard Gaussian distribution, allowing the contributions from different PMT strata to be combined in an additive fashion.

To test the effect of allele A, for instance, the following strata are informative:

**Mating**      **Informative Offspring Genotypes**

   A.A~A.x: A.A   vs   A.x

   A.x~A.x: A.A   vs         x.x

   A.x~x.x:      A.x      vs   x.y

   A.x~y.y:      A.y      vs   x.y

   A.x~A.y: A.A   vs         x.y

   A.x~x.y:      (A.x + A.y)   vs   (x.x + x.y)

   A.y~x.y:      (A.x + A.y)   vs   (x.y + y.y)

   A.x~y.z:      (A.y + A.z)   vs   (x.y + x.z)

The informative children A.A in the A.x~A.x and A.x~A.y strata differ from the 'non-A' (x.x or x.y) counterparts by two alleles. As in [24], this will be accounted for by assigning twice the weight to these strata, rather than assuming that the effects of the two alleles transmitted to the same child are independently observed.

With more than two alleles, several comparisons could be made within the A.x~A.y stratum, e.g.,

**Mating**      **Informative Offspring Genotypes**

   A.x~A.y: (A.A + A.y)   vs   (A.x + x.y)

   A.x~A.y: (A.A + A.x)   vs   (A.y + x.y)

In both cases, the term to the left (A.A or A.x/A.y) would have more A alleles than the corresponding term on the right (A.x/A.y or x.y, respectively). Averaging across these comparisons yields the same term (A.x+A.y)/2 on both sides which, as the heterozygous children born to two heterozygous children in the biallelic case, can be ignored, resolving this seeming ambiguity. Again, stratification is essential for developing a sound statistical approach to deal with this complex situation.

**Figure 1:**
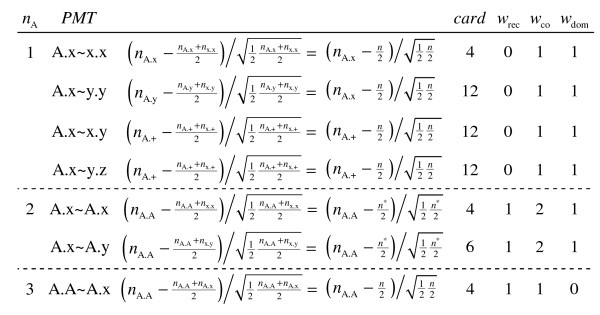
**Standardized residuals by parental mating type (PMT) with cardinality (card: number of combinations of non-A nucleotides x, y, and z per row) and weight (w: weight, depending on degree of dominance)**.

From column *card *in Figure 1, the maximum number of informative strata for the influence of a given allele is 3 × 4 + 3 × 12 + 6 = 54 out of the 105. Of course, if a particular PMT is not observed, the corresponding strata need not be included. Column *w *gives the weight to be applied to this stratum's contribution based on the number of alleles differing.

This stratification now allows for different degrees of 'dominance' to be considered. An allele is "dominant" or "recessive", if having a single copy confers the same risk as having two copies or no copy, respectively.

When both parents are heterozygous for the allele of interest, only those children are counted where both alleles are equal to or different from the allele of interest (n* = *n*_A. A _+ *n*_¬A.¬A_). Thus, among these strata, the effective sample size depends on the allele considered.

Using the results of [30], the strata's contributions can be combined into an allele-specific test statistic by forming a single test statistic from the sum of the effect estimates and their variances, respectively, e.g.,

$$T_{\text{A}}=\frac{\sum_{x\neq y\neq z\in \left\{\text{C,G,T,X}\right\}}\sum_{PMT\in PMT\left(\text{A}\right)}w_{PMT}\left(n_{{\text{A}}^{+}|PMT}-n_{{\text{A}}^{-}|PMT}\right)}{\sqrt{\sum_{x\neq y\neq z\in \left\{\text{C,G,T,X}\right\}}\sum_{PMT\in PMT\left(\text{A}\right)}w_{PMT}^{2}\left(n_{{\text{A}}^{+}|PMT}+n_{{\text{A}}^{-}|PMT}\right)}}\sim_{as.}\text{N}\left(0,1\right)$$

where x is the subset of the 54 PMTs informative (relevant and not empty) for nucleotide A, while $n_{{\text{A}}^{+}|PMT}$ and $n_{A^{-}|PMT}$ states the number of subjects with a preponderance of A or non-A alleles, respectively within the stratum indicated (i.e., *n*_*A*. *A*_, *n*_*A*. *x*_, or *n*_*A*.+_, see above).

From the above, nucleotide-specific test statistics can be obtained by performing the following steps:

1) Select the PMTs where at least one parent is heterozygous for the nucleotide considered (Figure 1).

2) Among the strata where both parents are heterozygous for the nucleotide considered, eliminate the counts of children that are also heterozygous for this nucleotide.

3) By number of parental copies of the nucleotide of interest (1, 2, or 3), count the number of children with more (1 or 2) vs the number of children with less (0 or 1, respectively) alleles of this nucleotide.

4) Perform the stratified McNemar test [24] based on these six numbers and the weights assigned to each of the three categories of strata according to the degree of dominance to be considered (Figure 1).

### Software

Statistical analyses were done using S-PLUS 8.0  and the muStat library (available from  and ). Surface and cartoon representations were produced using PyMOL . The program VOLUMES (R. Esnouf., unpublished data) was used with a 1.4 Å radius probe to map the extent of the P4 pocket cavity.

## Results

Of 7333 genotyped subjects, 3178 children (1773 affected and 1405 unaffected) from 1352 families (1–15 children per family) had complete familial allele-type and disease status information available.

Sequence analysis identified 93 SNPs that suffice to characterize the differences between the 13 main allelic types of *HLA-DRB1*. As shown in Table 1, the relationship between nucleotides and allelic groups is complex. Thus, characterizing subjects by the 13 allelic groups (two-digit resolution) may not suffice to identify the genetic factors contributing to disease susceptibility (or resistance). For instance, for the second SNP (nucleotide position (N) 016), HLA-DRB1*04 (T) is uniquely different from all other groups (C), while the third SNP (N037) separates HLA-DRB1*01, *15, and *16 (A) from all other alleles (G). HLA-DRB1*09 is the only allelic group characterized by a single allele at each of the 93 SNPs – all other allelotypes have genetic variability, often with more than two alleles observed within each allelic group at a given SNP. The region between N256 and N308 is characterized by various allelotypes with three or four potential nucleotides per SNP.

**Table 1:** Nucleotides (N#: nucleotide number, #A: amino acid number) found in 94 HLA-DRB1 SNPs discriminating the 13 main two-digit allelic groups (rows).

Allelotype:		02		01	04	03	05		06		08	10	09	07
A#	N#	15	16			17/18	11	12	13	14				
-25	14	A	A	A	A	**G**	**G**	**G**	**G**	**G**	**G**	**G**	A	A
-24	16	C	C	C	**T**	C	C	C	C	C	C	C	C	C
-17	37	A	A	A	**G**	**G**	**G**	**G**	**G**	**G**	**G**	**G**	**G**	**G**
-16	41	C	C	C	C	**T**	**T**	**T**	**T**	**T**	**T**	**T**	C	C
-16	42	G	G	G	**T**	**T**	**T**	**T**	**T**	**T**	**T**	**T**	**T**	**T**
-1	85	T	**G**	**G**	**G**	**G**	**G**	**G**	**G**	**G**	**G**	**G**	**G**	**G**
-1	86	C	C	C	C	C	C	C	C	C	C	C	C	C
-1	87	T	T	T	T	T	T	T	T	T	T	T	T	T
4	97	C	C	C	C	**A**	**A**	**A**	**A**	**A**	**A**	**A**	C	C
4	98	G	G	G	G	G	G	G	G	G	G	G	**A**	**A**
8	109	C	C	**T**	**T**	**T**	**T**	**T**	**T**	**T**	**T**	**T**	**T**	C
9	112	T	T	T	**G**	**G**	**G**	**G**	**G**	**G**	**G**	**G**	**A**	T
10	115	C	C	C	C	**T**	**T**	**T**	**T**	**T**	**T**	**G**	C	C
10	117	G	G	G	G	**C**	**C**	**C**	**C**	**C**	**C**	G	G	G
11	118	C	C	C	**G**	**T**	**T**	**T**	**T**	**T**	**T**	**G**	**G**	**G**
11	119	C	C	**T**	**T**	C	C	C	C	C	C	**T**	**A**	**G**
12	122	A	A	A	A	**C**	**C**	**C**	**C**	**C**	**C**	A	A	A
13	124	A	A	**T**	**C**	**T**	**T**	**G**	**T**	**TG**	**G**	**T**	**T**	**T**
13	125	G	G	**T**	**A**	**C**	**C**	G	**C**	**CG**	G	**T**	**T**	**A**
13	126	G	G	**T**	**T**	**T**	**T**	**T**	**T**	**T**	**T**	**T**	**T**	**T**
14	127	G	G	G	G	G	G	G	G	G	G	G	G	**A**
14	129	G	G	**A**	G	G	G	G	G	G	G	G	G	G
16	133	C	C	C	C	C	C	**T**	C	**CT**	**T**	C	C	C
19	144	T	T	T	**C**	T	T	T	T	T	T	**C**	**C**	**C**
26	164	T	T	T	T	**AT**	T	T	T	T	T	T	**A**	T
26	165	C	C	**G**	C	C	C	**A**	C	C	C	**G**	**T**	C
28	169	G	G	G	G	G	G	G	G	G	G	G	**C**	G
28	171	C	C	**A**	C	**CG**	C	**G**	**CG**	**CG**	C	**A**	C	**A**
30	175	T	T	T	T	T	T	**C**	T	T	T	**C**	**G**	**C**
30	176	A	A	**G**	A	A	A	A	A	A	A	**G**	**G**	**T**
31	178	T	T	**A**	T	T	T	T	T	T	T	**G**	**A**	T
32	181	T	T	T	T	**C**	**CT**	**C**	**CT**	**CT**	**CT**	**C**	T	T
33	184	A	A	A	**C**	A	A	A	A	A	A	A	A	A
34	189	G	G	**A**	**A**	G	**AG**	G	**AG**	**AG**	**AG**	**A**	**A**	G
37	196	T	T	T	T	**AT**	**AT**	**CT**	**AT**	**AT**	T	T	**A**	T
37	197	C	C	C	**AC**	**AT**	**AT**	**T**	**AT**	**AT**	**AT**	**A**	**A**	**T**
38	199	G	G	G	G	G	G	**C**	G	G	G	G	G	G
38	200	T	T	T	T	T	T	T	T	T	T	**C**	T	T
47	227	T	**AT**	**A**	**AT**	**AT**	**AT**	T	**AT**	**AT**	**A**	**A**	**A**	**A**
53	246	G	G	G	G	G	G	G	G	G	G	G	G	**A**
57	256	G	G	G	**AG**	G	G	G	**AG**	**AG**	**AG**	G	G	G
57	257	A	A	A	**AG**	A	A	**AT**	**ATG**	**AC**	**ATG**	A	**T**	**T**
57	258	**CT**	**C**	T	**CT**	T	T	**CT**	**CT**	**CT**	**CT**	T	**C**	**C**
58	260	C	C	C	C	C	**A**	C	C	C	C	C	C	C
58	261	**CT**	T	**C**	**CTG**	**CG**	**G**	**C**	**CT**	**CTG**	**CG**	**C**	**C**	**C**
60	265	T	T	T	T	T	T	T	T	**CT**	T	T	T	T
60	266	A	A	A	A	A	A	**AC**	**AC**	A	A	A	**C**	**C**
60	267	A	A	A	A	A	A	A	A	A	A	A	A	A
67	286	**AC**	**ACT**	**AC**	**ACT**	**C**	**ACT**	**AT**	**ACT**	**ACT**	**AT**	**C**	**T**	A
69	294	**AG**	**A**	G	**AG**	G	**AG**	**A**	**A**	**AG**	**A**	G	G	G
70	295	**CG**	**G**	C	**CG**	C	**CG**	**G**	**G**	**CG**	**G**	C	C	**G**
70	296	A	A	A	A	A	A	A	A	**AG**	A	**G**	**G**	A
70	297	G	**C**	G	**CG**	G	**CG**	**C**	**C**	**CG**	**C**	G	G	**C**
71	298	**AG**	**A**	**AG**	**AG**	**A**	**AG**	**A**	**AG**	**AG**	**A**	**A**	**A**	**A**
71	299	C	**G**	**ACG**	**AG**	**A**	**AG**	**G**	**AG**	**AG**	**G**	**G**	**G**	**G**
72	303	G	**CG**	G	G	G	G	**CG**	**CG**	G	G	**CT**	G	G
73	305	C	C	C	C	**G**	C	C	C	C	C	C	C	**G**
74	307	G	G	G	**CG**	**C**	**CG**	G	**CG**	**CG**	**CG**	G	G	**C**
74	308	C	C	C	**ACT**	**AG**	**ACT**	C	**CT**	**ACT**	**CT**	C	**A**	**A**
77	317	C	C	**AC**	**AC**	**A**	C	C	C	**AC**	C	C	C	C
77	318	C	C	**CT**	C	**CT**	C	C	C	C	C	C	C	C
78	320	A	A	A	A	A	A	A	A	A	A	A	**T**	**AT**
78	321	C	C	**CT**	C	C	**CT**	**CT**	C	**CT**	C	C	**G**	**CG**
85	341	T	T	**CT**	T	T	**CT**	**CT**	T	T	T	T	T	T
86	344	**TG**	**T**	**TG**	**TG**	**TG**	**TG**	G	**TG**	**TG**	**TG**	**TG**	**T**	**T**
90	357	A	A	A	**AG**	A	**AG**	A	**AG**	A	**AG**	A	A	A
93	365	C	C	C	C	C	C	C	C	C	C	C	**A**	C
95	372	C	C	**T**	C	C	C	C	C	C	C	**T**	C	C
96	373	C	C	**G**	**T**	C	C	C	C	C	C	C	C	C
96	374	A	A	A	A	A	A	A	A	A	A	A	A	A
96	375	A	A	**G**	**T**	**T**	**T**	**T**	**T**	**T**	**T**	A	**T**	**T**
98	379	A	A	A	**G**	A	A	A	A	A	A	A	**G**	**G**
101	388	G	G	G	G	G	G	G	G	G	G	G	G	G
101	389	T	T	T	T	T	T	T	T	T	T	T	T	T
101	390	A	A	**G**	**G**	**G**	**G**	**G**	**G**	**G**	**G**	**G**	**G**	**G**
104	397	T	T	T	**G**	T	T	T	T	T	T	T	**G**	**G**
106	405	C	C	C	C	C	C	C	C	C	C	C	**T**	**T**
114	429	C	C	C	C	C	C	C	C	**CG**	C	C	C	C
117	438	C	C	C	C	**T**	**T**	**T**	**T**	**T**	**T**	**T**	C	C
120	446	G	G	G	**A**	G	G	G	G	G	G	**A**	G	G
133	484	C	C	C	C	C	C	C	C	C	C	C	C	C
133	485	T	T	**G**	**G**	**G**	**G**	**G**	**G**	**G**	**G**	**G**	**G**	**G**
133	486	G	G	G	G	G	G	G	G	G	G	G	G	G
134	489	C	C	C	C	**T**	**T**	**T**	**T**	**T**	**T**	C	C	C
140	505	G	G	G	**A**	**A**	**A**	**A**	**A**	**A**	**A**	**A**	G	G
142	511	A	A	**G**	**G**	**G**	**G**	**G**	**G**	**G**	**G**	**G**	**G**	**G**
142	512	T	T	T	T	T	T	T	T	T	T	T	T	T
142	513	G	G	G	G	G	G	G	G	G	G	G	G	G
145	522	A	A	A	A	A	A	**G**	A	A	A	**G**	A	A
149	534	G	G	G	G	**C**	**C**	**C**	**C**	**C**	**C**	G	G	G
152	543	C	C	**T**	C	C	C	C	C	C	C	C	C	C
166	583	C	C	C	C	C	C	C	C	C	C	C	C	C
166	584	G	G	G	G	G	G	G	G	G	G	**A**	G	G
166	585	A	A	**G**	**G**	**G**	**G**	**G**	**G**	**G**	**G**	**G**	**G**	**G**
169	594	G	G	G	G	G	G	G	G	G	G	G	**A**	**A**
179	624	C	C	**T**	C	C	C	C	C	C	C	**T**	**T**	**T**
180	625	G	G	G	**C**	G	G	G	G	G	G	G	G	G
181	629	C	C	C	C	C	C	C	C	C	C	**T**	**T**	**T**
181	630	A	A	**G**	**G**	A	A	A	A	A	A	**G**	**G**	**G**
190	656	A	A	A	A	A	A	A	A	A	**T**	A	A	A
207	707	T	T	**C**	**C**	T	T	T	T	T	T	T	T	T
218	739	C	C	C	C	C	C	C	C	C	C	C	C	**T**
232	783	A	A	A	A	A	A	A	A	A	A	**C**	A	A
234	789	C	C	C	C	**G**	**G**	**G**	**G**	**G**	C	C	C	C

#Cases:		3994	208	1396	1982	1873	1068	146	1519	243	408	80	98	1653

Note that many SNPs have more than two nucleotides and some SNPs have several nucleotides for the same allelic group. For instance, Nucleotide 257 can be either A or T within allelotype *12 and A, T, or G within allelotype *13. Given the high prevalence of the HLA-DRB1*15 (15) allele, the nucleotide found in this group of alleles was used as the reference, unless several nucleotides were found in the alleles constituting the HLA-DRB1*15 group.

This would render current statistical analysis strategies, comparing one allelic group against all others, highly inefficient. To resolve this conundrum, we extended a recently developed statistical approach that allowed us to assess at each of the 93 loci the association (controlled for population admixture) with each individual nucleotide (A, C, G, T, and deletion). The test statistics for this analysis are shown in Table 2 (left side).

**Table 2:** Test statistics for specific alleles at any of the 93 SNPs increasing (positive) or decreasing (negative) the risk of MS.

	#N	#A	A	C	G	T		A	C	G	T	
1	14	-25	6.723		-6.723		1231	0.506		-0.506		995
2	16	-24		4.485		-4.485	747		0.512		-0.512	593
3	37	-17	**12.563**		***-12.563***		1249	1.774		-1.774		1018
4	41	-16		6.723		-6.723	1231		0.506		-0.506	995
5	42	-16			**12.563**	***-12.563***	1249			1.774	-1.774	1018
6	**85**	**-1**			***-15.587***	**15.587**	1139			***-2.985***	**2.985**	926
7	97	4	-6.723	6.723			1231	-0.506	0.506			995
8	98	4	-3.206		3.206		696	-1.308		1.308		547
9	109	8		11.817		-11.817	1311		1.629		-1.629	1028
10	112	9	-1.313		-9.607	9.830	1340	0.354		-0.829	0.776	1066
11	115	10		6.723	-1.000	-6.565	1245		0.506	0.316	-0.562	1004
12	117	10		-6.565	6.565		1236		-0.562	0.562		993
13	118	11		**12.563**	-6.002	-6.565	1544		1.774	-1.323	-0.562	1245
14	119	11	-1.313	7.597	-2.961	-6.254	1360	0.354	2.093	-1.418	-1.354	1073
15	122	12	6.565	-6.565			1236	0.562	-0.562			993
16	**124**	**13**	**14.806**	-5.027	-1.172	-9.371	1425	**3.079**	-0.726	0.884	***-2.514***	1133
17	**125**	**13**	-6.121	-5.444	**13.465**	-4.346	1507	-1.794	-0.856	**3.240**	-1.205	1225
18	**126**	**13**			**15.117**	***-15.117***	1171			**2.781**	***-2.781***	961
19	127	14	-2.961		2.961		657	-1.418		1.418		528
20	129	14	-3.750		3.750		544	-1.403		1.403		455
21	133	16		1.172		-1.172	236		-0.884		0.884	155
22	144	19		-6.002		6.002	1118		-1.323		1.323	897
23	164	26				0.156	41	0.730			-0.730	30
24	165	26		4.739	-3.887	-1.313	663	1.508	0.732	-1.289	0.354	531
25	169	28		-1.313	1.313		58		0.354	-0.354		32
26	171	28	-4.609	4.964	-1.732		395	-0.967	0.981	0.000		310
27	175	30		-3.790	-1.313	4.009	770		-0.885	0.354	0.792	617
28	176	30	5.203		-4.158	-2.961	1055	1.941		-1.169	-1.418	857
29	178	31	-4.029		-1.000	4.158	605	-1.277		0.316	1.169	505
30	181	32		-1.742		1.742	309		0.000		0.000	255
31	184	33	4.485	-4.485			747	0.512	-0.512			593
32	189	34	-5.998		5.998		425	-0.372		0.372		348
33	196	37					18					9
34	197	37	-1.897	3.130		-2.629	71	-0.333	1.386		-1.361	58
35	199	38		-2.438	2.438		88		1.508	-1.508		44
36	200	38		-1.000		1.000	36		0.316		-0.316	40
37	227	47	-4.743			4.743	116	-2.008			2.008	87
38	246	53	-2.961		2.961		657	-1.418		1.418		528
40	257	57	4.879			-4.879	208	**3.048**			***-3.048***	153
41	258	57		-1.121		1.121	29		-1.291		1.291	11
42	260	58	-4.929	4.929			461	0.246	-0.246			390
43	261	58		-0.775	0.000	1.134	9		0.258	0.000	-0.577	9
45	266	60	2.863	-2.863			488	2.066	-2.066			373
46	286	67	-0.447	0.447			5	-0.577	0.577			3
47	294	69	-0.956		0.956		67	-0.117		0.117		49
48	295	70		2.656	-2.656		69		0.289	-0.289		39
49	296	70	1.877		-1.877		92	-0.367		0.367		67
50	297	70		-6.363	6.363		428		-1.261	1.261		300
52	299	71	-0.884	4.447	-4.196		159	-0.717	**2.535**	-2.137		96
54	305	73		2.218	-2.218		1086		1.724	-1.724		903
55	307	74		-3.558	3.558		203		***-2.571***	**2.571**		139
56	308	74	-2.333	2.333			72	-1.477	1.477			55
57	317	77	-1.191	1.191			273	-0.931	0.931			186
59	320	78	1.121			-1.121	39	-0.426			0.426	22
60	321	78		2.183	-2.183		17		0.632	-0.632		10
62	344	86			-1.414	1.414	2			0.000	0.000	0
64	365	93	-1.313	1.313			58	0.354	-0.354			32
65	372	95		3.887		-3.887	566		1.289		-1.289	476
66	373	96		6.168	-3.750	-4.485	1085		1.428	-1.403	-0.512	880
67	**375**	96	**14.920**		-3.750	***-12.385***	1386	**2.799**		-1.403	-1.818	1135
68	379	98	5.914		-5.914		1106	1.400		-1.400		887
69	**390**	101	**15.117**		***-15.117***		1171	**2.781**		***-2.781***		961
70	397	104			-5.914	5.914	1106			-1.400	1.400	887
71	405	106		3.206		-3.206	696		1.308		-1.308	547
73	438	117		6.723		-6.723	1231		0.506		-0.506	995
74	446	120	-4.595		4.595		769	-0.422		0.422		609
75	**484**	133			***-15.117***	**15.117**	1171			***-2.781***	**2.781**	961
76	489	134		6.565		-6.565	1236		0.562		-0.562	993
77	505	140	-9.607		9.607		1325	-0.829		0.829		1057
78	**511**	142	**15.117**		***-15.117***		1171	**2.781**		***-2.781***		961
79	522	145	2.573		-2.573		121	-1.342		1.342		82
80	534	149		-6.565	6.565		1236		-0.562	0.562		993
81	543	152		3.750		-3.750	544		1.403		-1.403	455
82	584	166	-1.000		1.000		36	0.316		-0.316		40
83	**585**	166	**15.117**		***-15.117***		1171	**2.781**		***-2.781***		961
84	594	166	-3.206		3.206		696	-1.308		1.308		547
85	624	179		5.203		-5.203	1003		1.941		-1.941	816
86	625	180		-4.485	4.485		747		-0.512	0.512		593
87	629	181		3.348		-3.348	718		1.199		-1.199	581
88	630	181	7.597		-7.597		1248	2.093		-2.093		999
89	656	190	-0.450			0.450	180	-0.616			0.616	132
90	707	207		-6.168		6.168	1032		-1.428		1.428	823
91	739	218		2.961		-2.961	657		1.418		-1.418	528
92	783	232	1.000	-1.000			36	-0.316	0.316			40
93	789	234		6.813	-6.813		1207		0.771	-0.771		989

Left side: analysis of cases, right side: analysis of unaffected siblings.

Traditionally, family-based association studies utilize information from affected children only. With MS, however, the unaffected siblings of cases can be reasonably assumed to have similar genetic and environmental risk factors. Thus, one can analyze the siblings in the same fashion as the controls, after reversing the role of putative protective and risk alleles. If a nucleotide at a particular locus confers a risk, its absence should confer protection. The right side of Table 2 shows that the genetic constellations seen in the siblings, in fact, closely resemble the results seen in the cases, thereby validating the results.

To be asymptotically equivalent with the TDT [25], the stratified McNemar test [24], by default, assumes a co-dominant model, doubling the weight of the homozygous children born to two heterozygous parents. For dominant and recessive alleles, homozygous children (for the risk allele or the wild type, respectively) carry either no or the same risk, respectively, as heterozygous children, Thus, by assigning zero weight to some and equal weights to other strata, one can shift the power towards recessive and dominant models, respectively. The results under the dominant, but not the recessive model (data not shown) were similar to the results under the co-dominance model, suggesting that individual alleles contribute substantially to risk.

Based on these results, candidate loci for MS risk are (N = nucleotide position, A = Amino acid position):

**Position**            **Nucleotide(s)**      **Allelotype Carrying Risk Nucleotide**

N085:   T   HLA-DRB1*15

N124-126/A013:   AGG   HLA-DRB1*15/16

N375/A096:   A   HLA-DRB1*10/15/16

N390, 485, 511, 585/A101-142:   A, T, A, A   HLA-DRB1*15/16

**Figure 2:**
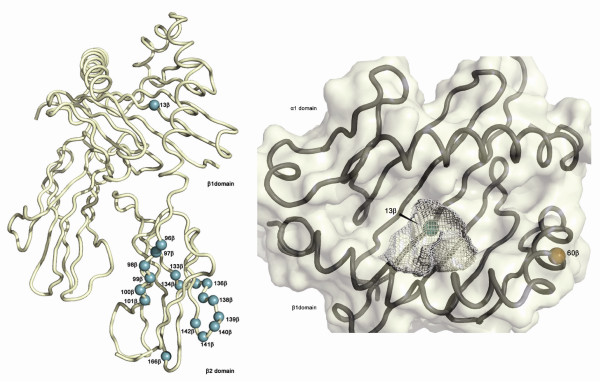
Position of candidate risk polymorphic residues in HLA-DRB1*1501. a. Cartoon representation of HLA-DRB1 (PDB ID 1BX2) in which the Cα atoms of candidate risk polymorphic residues (Table 2) are represented as small spheres (green). The β1 and β2 domains are labeled. b. Surface and cartoon representation of HLA-DRB! viewed from above the binding groove. The three-dimensional arrangement of the P4 pocket is shown as a grey mesh. The Cα atoms of the polymorphic residues 13 β (green) at the base of the P4 pocket and residue 60 β (orange) are represented as small spheres.

Thus, the sequence variants of HLA-DRB1*15 that increase MS risk can be narrowed down to these regions (Figure 2). N085 is in the promoter region, A013 is part of the antigen presentation P4 pocket, while A133-142 are part of the CD4 binding region [29]. (The region from #33 (N196/A37) to #64 (N365/A93) has more variability than the 13 categories can explain.)

## Discussion

Multiple sclerosis is unambiguously associated with the MHC class II region and this gene exerts the strongest genetic effect on the risk of developing the disease [3]. We have shown that A013 is the key amino acid in defining the risk of *HLA-DRB1*, yet no molecular or functional explanation can be given for the dominant-negative effects of *14 and *11, the complementary effects of *08, and the protective nature of *01 and *10 in the presence of *15. It has been speculated that poor engagement of the encephalitogenic peptide in the context of *14 acts to alter the immune response in a dominant-negative manner and thereby reduce the effect of *15 [20]. Another explanation is that *14 binds one or more peptides that can delete autoreactive T cells.

In this study, we performed the first empirical investigation of the nucleotide sequence of *HLA-DRB1 *with respect to determining disease risk. The statistical analysis employed in this study differs fundamentally from previous analyses conducted in that it is based on the actual nucleotides at a SNP, rather than HLA allelotypes. In other words, it allows at each locus the cases (or controls) to be categorized differently according to the nucleotides observed. In principle, we might have done the same analysis based on individual sequence data, irrespective of the two- (four- or six-) digit allele categories. In fact, using individual sequence data would avoid the ambiguities which reduced the sample size to fewer than 300 for the SNPs between N196 and N372 (Table 2) due to some allelotypes having many different nucleotides at these loci.

For amino acid 13, for instance, we count cases with unambiguous parental mating type for the following *HLA-DRB1 *allelotypes (see Table 3):

**Table 3:** 

**Nucleotide**	**N124**	**N125**	**N126**
**A:**	15, 16	04, 07	-
**C:**	04	03, 11, 13	-
**G:**	12, 08	15, 16, 08	15, 16
**T:**	01, 03, 11, 13, 10, 09, 07	01, 10, 09	01, 04, 03, 11, 12, 13, 14, 08, 10, 09, 07d
**ambiguous:**	14	14	

(*numbers indicate HLA-DRB1 allelotype*)

As the categories are refined or individual sequence data becomes available, ambiguous cases will become fewer or avoided altogether, respectively.

As in the case of case-control studies [31], counting alleles transmitted to cases, as suggested by [25] in the widely used Transmission-Disequilibrium-Test (TDT) has recently been proven to result in a suboptimal analysis strategy even in the case of bi-allelic parents [24], because the TDT's variance estimate is based, in part, on the counts of non-informative heterozygous children born to two heterozygous parents. As with Student's t- vs. the Gauss' z-test (which are also asymptotically equivalent), this additional variance would need to be accounted for. The stratified McNemar test [24], in contrast, avoids this problem by replacing 2 × (*n*_P. P _+ *n*_P. Q _+ *n*_Q. Q_) in the denominator by 4 × (*n*_P. P _+ *n*_Q. Q_). For tri- or tetra-allelic parents, stratification is even more important, because it also leads to novel analysis strategies.

Barcellos *et al. *[20] have suggested that amino acid 60 accounts for the protective effect of the HLA-DRB1*14 allele, because this locus is close to the P4 binding pocket and HLA-DRB1*1401 encodes histidine (CAC), while HLA-DRB1*1701, *0401, *0801 and *1501 all encode tyrosine (TAC). Upon close inspection of the SNP profiles, however, the observed relationship between HLA-DRB1*15/*15, HLA-DRB1*15/*08 and HLA-DRB1*14 seems to be more complex.

Among the 4-digit *HLA-DRB1 *allelotypes, the majority of alleles code TAC for tyrosine, as does HLA-DRB1*15 (all 4-digit allelotypes).

**Codon**      Four digit HLA-DRB1*14 allelotype carrying codon

   TAC (14xx): 02,03,05,06,09,11,12,13,14,15,17,18,19,20,21,23,24,27,29,30,33,

         36,37,40,41,42,43,44,45,36,47,51,52,56,57,59,63,65,67,76,77

   CAC (14xx): 01,04,07,08,10,16,22,25,26,28,31,32,34,35,38,39,49,50,53,54,55,

         58,60,61,62,68,69,70,71,72,73,74,75

   TCC (14xx): 48,64

In the population of [32], 30% of the population were genotyped at the 4-digit level. Among them, 82% were *1401 and 9% were *1404. If one assumes that these 30% were representative and that the same proportion holds for our population, H at P60 could exert its "protective" effect in about 90% of all 14xx subjects. However, the purported disease risk increasing TAC codon is also seen in *0101, *0103, *0104, *0110, and *0111. Thus, if this amino acid should cause *08 to have its special role in *15/*08 heterozygous subjects, this effect should be seen with other alleles, yet this is not the case [11,12].

For amino acid 13, instead, *15 (all K) is disjoint from both *08 (all G/glycine) and *14 (mostly S/serine)

**Codon**      ***HLA-DRB1 *allelotype carrying codon**

   AGG/K (15xx): (all)

   GGT/G (08xx): (all)

   TCT/S (14xx): 01,02,03,05,06,07,08,09,12,13,14,16,17,18,19,20,21,22,23,24,25,

         26,27,32,33,34,35,36,37,38,39,40,41,42,43,44,45,46,47,48,49,51,

         53,54,55,56,58,59,60,62,63,64,65,66,67,69,70,72,74,75,77

   GGT/G (14xx): 04,11,15,28,29,30,50,52,61,68,71,73,76,

   CAT/H (14xx): 10,31,57

From this data, it seems unlikely that histidine at amino acid 60 contributes substantially to the protective effect of this haplotype. Instead, amino acid 13 emerges as a more likely explanation for a disease association gradient [32]. It is the only amino acid that shows sufficient variation to explain a hierarchy of disease associated alleles, raising the possibility that TCT/S is, in fact, associated with protection, while GTT/G is associated with increased risk. Being located right in the center of the P4 binding pocket, amino acid 13 is a residue that is potentially in contact with presented peptides [29,32]. Another interesting finding of this paper is the implication that the promoter region, may play an important role. This finding lends notion to the idea of regulatory variants contributing to MS risk. As we have previously shown [33], *HLA-DRB1 *(and therefore amino acid 13) cannot fully explain the MHC class II associated MS risk and these nearby variants remain to be uncovered.

It has been argued that "low resolution allele grouping [...] maximize [s] statistical power" [20', p. 2821] when comparing one group against all others. When increasing the sample size comes at the expense of increasing within group variance, however, statistical power often suffers. Let us assume that group *02 had not been separated into *15 (3994 cases) and *16 (208) cases. Then, according to Table 4, 208 'G' cases would have been added to the 3,994 'T' cases to a total of 4202, but the size of the 'G' group would have been reduced from 10,674 to 10,466. Even though the number of informative trios would slightly increase (from 1139 to 1171, see Table 2), the test statistic would drop from 11.022 to 10.689. The proposed statistical test, in contrast, utilized the information obtained through high resolution allelotyping (or sequencing) to increase group sizes by combining allelotypes with the same nucleotide at a given locus.

**Table 4:** Variation of genotypes for the amino acids marked in Table 2.

Allelotype:		02		01	04	03	05		06		08	10	09	07
A#	N#	15	16			17/18	11	12	13	14				
-1	85	**T**	G	G	G	G	G	G	G	G	G	G	G	G
-1	86	C	C	C	C	C	C	C	C	C	C	C	C	C
-1	87	T	T	T	T	T	T	T	T	T	T	T	T	T
13	124	**A**	**A**	T	C	T	T	G	T	TG	G	T	T	T
13	125	**G**	**G**	T	A	C	C	**G**	C	C**G**	G	T	T	A
13	126	**G**	**G**	T	T	T	T	T	T	T	T	T	T	T
60	265	T	T	T	T	T	T	T	T	CT	T	T	T	T
60	266	A	A	A	A	A	A	AC	AC	A	A	A	C	C
60	267	A	A	A	A	A	A	A	A	A	A	A	A	A
96	374	A	A	A	A	A	A	A	A	A	A	A	A	A
96	375	**A**	**A**	G	T	T	T	T	T	T	T	A	T	T
101	388	G	G	G	G	G	G	G	G	G	G	G	G	G
101	389	T	T	T	T	T	T	T	T	T	T	T	T	T
101	390	**A**	**A**	G	G	G	G	G	G	G	G	G	G	G
133	484	C	C	C	C	C	C	C	C	C	C	C	C	C
133	485	**T**	**T**	G	G	G	G	G	G	G	G	G	G	G
133	486	G	G	G	G	G	G	G	G	G	G	G	G	G
142	511	**A**	**A**	G	G	G	G	G	G	G	G	G	G	G
142	512	T	T	T	T	T	T	T	T	T	T	T	T	T
142	513	G	G	G	G	G	G	G	G	G	G	G	G	G
166	583	C	C	C	C	C	C	C	C	C	C	C	C	C
166	584	G	G	G	G	G	G	G	G	G	G	A	G	G
166	585	**A**	**A**	G	G	G	G	G	G	G	G	G	G	G

#Cases:		3994	208	1396	1982	1873	1068	146	1519	243	408	80	98	1653

(Rare observations, such as TCC in only two out of 77 14xx subcategories are omitted)

As we have demonstrated above, introducing the concept of stratification by parental mating type has not only quantitative advantages [24] over the original more simplistic approach, but also qualitatively different results for SNPs with more than two alleles in the population. As more data are collected, the proposed method could even be extended to address the difference between association (*in trans*) and protein function (*in cis*). To do so, one could categorize filial genotypes by pairs of amino acids, rather than nucleotides, so that differences in nucleotides coding for the same protein would be ignored. Of course, to ensure that genetic confounders are eliminated, parental mating types should still be defined based on the genetic code, rather than the amino acid for which it is coding. While this strategy is based on a large number of possible combinations between three-nucleotide parental mating types and combinations of filial amino acids, the number of combinations observed at each locus is likely to be substantially smaller.

## Conclusion

In conclusion, an extended statistical approach allowed us to identify A013 at the center of the P4 pocket of HLA-DRB1 as a potentially important (although unlikely exclusive) risk factor for MS.

## Competing interests

The authors declare that they have no competing interests.

## Authors' contributions

KMW and GCE conceived and designed the experiments. SVR, RM, DAD, ADS and KMW performed the experiments. SVR, GCE and KMW analyzed the data and wrote the paper.

## Pre-publication history

The pre-publication history for this paper can be accessed here:


